# Prognostic significance of 4R lymph node dissection in patients with right primary non-small cell lung cancer

**DOI:** 10.1186/s12957-022-02689-w

**Published:** 2022-07-01

**Authors:** Di Zhou, Dongsheng Yue, Zhenfa Zhang, Pengfei Tian, Yingnan Feng, Zuo Liu, Bin Zhang, Meng Wang, Xiaoliang Zhao, Changli Wang

**Affiliations:** Department of Lung Cancer Surgery, Tianjin Medical University Cancer Institute and Hospital, Binshui Road, Tianjin, China

**Keywords:** Non-small cell lung cancer, Right primary lung cancer, Station 4R lymph nodes

## Abstract

**Background:**

To investigate the prognostic significance of station 4R lymph node (LN) dissection in patients who underwent operations for right primary non-small cell lung cancer (NSCLC).

**Methods:**

We performed a retrospective study involving patients with right primary NSCLC who received lobotomy or pneumonectomy with mediastinal LN dissection between January 2011 and December 2017. Propensity score matching was performed. Disease-free survival (DFS) and overall survival (OS) were compared between patients with and without station 4R dissection.

**Results:**

Our study included 2070 patients, with 207 patients having no station 4R dissection (S4RD− group) and 1863 patients having station 4R dissection (S4RD+ group). The 4R LN metastasis rate was 13.4% (142/1748), higher than that for other mediastinal LN metastases. Compared with the S4RD− group, the S4RD+ group had higher 5-year DFS (48.1% vs. 39.1%, *P* = 0.009) and OS (54.4% vs. 42.8%, *P* = 0.025). Station 4R dissection was an independent risk factor for DFS (odds ratio, OR, 1.28, 95% confidence interval, CI, 1.08–1.64, *P* = 0.007) and OS (OR 1.31, 95% CI 1.04–1.63, *P* = 0.018). Patients with adjuvant chemotherapy had a better prognosis after station 4R dissection than those without adjuvant chemotherapy (57.4% vs. 52.3%, *P* = 0.006). The 5-year OS in the station 4R metastasis group was lower than that in the station 4R non-metastasis group (26.9% vs. 44.3%, *P* = 0.006) among N2 patients. The 5-year OS of the single-station 4R metastasis group was lower than that of the single-station 7 metastasis group (15.7% vs. 51.6%, *P* = 0.002).

**Conclusions:**

Station 4R metastasis was the highest among all the mediastinal station metastases in right primary NSCLC patients. Station 4R dissection can improve the prognosis and should be recommended as a routine procedure for these patients.

## Background

Lung cancer is the leading cause of cancer-related deaths worldwide [1]. Non-small cell lung cancer (NSCLC) is the most common type of lung cancer [2]. The mainstay treatment for NSCLC is surgical resection [3]. During the operation, it is essential to remove the tumor as well as involved lymph nodes (LNs). However, preoperative imaging determination of mediastinal LN metastasis can be difficult, with a sensitivity and specificity of only 57% and 82%, respectively [4, 5]. Therefore, mediastinal lymph node dissection (MLND) was proposed as an essential step during the surgical treatment of NSCLC. MLND can provide important prognostic and therapeutic value for patients with NSCLC. Current guideline recommends that MLND should remove at least three mediastinal nodal stations, including station 7 [6]. However, the application of MLND in clinical practice remains controversial, since some studies reported increased operating time, prolonged hospital stays, and potentially high mortality with the procedure [7].

In the mediastinum, station 4R LNs are surrounded by complicated structures, such as the trachea, bronchus, pulmonary arteries, superior vena cava, and azygos. Studies have found that the station 4R LN metastasis rate was high but the resection rates varied between 64.5 and 87.1% [8–11]. Few studies have been conducted to study the association between station 4R LN dissection and patient prognosis. Therefore, we performed the present study to investigate station 4R metastasis and its association with the prognosis in patients with right primary NSCLC.

## Methods

### Study design and participants

We performed a retrospective study and reviewed the data for patients with right primary NSCLC who visited Tianjin Medical University Cancer Institute and Hospital, China, between January 2011 and December 2017. The study protocol was approved by the hospital ethics committee.

Patients were included if they had right primary NSCLC and received lobectomy or pneumonectomy with MLND. They were excluded if they (1) had metastatic lung cancer, (2) received pulmonary segment or pulmonary wedge resection, (3) received LN dissection but did not meet the criteria for MLND [6], (4) received preoperative neoadjuvant chemotherapy or radiotherapy, or (5) had incomplete medical records.

All patients underwent routine preoperative evaluations. Lobectomy or pneumonectomy with MLND was performed with thoracotomy or thoracoscopy by experienced thoracic surgeons. Based on the recommended LN clearance criteria during the radical surgical resection of lung cancer proposed by the International Association for the Study of Lung Cancer (IASLC) and the relevant guidelines [6, 12], at least three mediastinal LN stations, including the subcarinal LNs, as well as stations 10, 11, and 12 LNs, were routinely removed. The station 13 and 14 LNs were also removed along with the adjacent lung tissue but no pathologic examinations were performed on the tissues. The resected lung cancer tissue and LNs were evaluated by two experienced pathologists. All patients were staged according to the 8th edition TNM criteria [13].

### Postoperative follow-up evaluations

All patients were followed up postoperatively. The follow-up outcomes were obtained from the hospital medical records. Patients received a chest X-ray or computed tomography (CT) scan 3–4 months after the surgery. Afterward, they were followed-up in the clinic every 4–6 months for the first 2 years and every 6–12 months for the next 3–5 years. After 5 years, patients were followed-up once a year. During the follow-up visits, patients received routine examinations, including laboratory tests, computed tomography (CT), magnetic resonance imaging (MRI), or positron emission tomography–computed tomography (PET/CT) scans when necessary.

The primary end point was the overall survival (OS) from the time of surgery to death or December 31, 2020. The secondary end point was the disease-free survival (DFS) from the time of surgery to cancer recurrence, metastasis, or the end of the study.

### Propensity score matching

Propensity score matching (PSM) was performed to minimize the influence from confounding variables [14]. The propensity score was calculated for each patient based on a propensity score model that included every possible covariate, such as age, gender, smoking history, surgical approaches, tumor location, adjuvant chemotherapy, histological type, pT stage, and pN stage. The patients were matched with the Nearest Neighbor method with caliper restrictions in R (version 4.0.4) using the Matchit package (Version 4.1.0).

### Statistical analysis

Patients were assigned to either the S4RD− group (without 4R dissection) or the S4RD+ group (with 4R dissection). The categorical variables were compared with the chi-square test. A multivariate logistic regression model was constructed to evaluate the relationship between station 4R metastasis and risk variables. The reverse Kaplan-Meier method was used to calculate the median follow-up time. Survival outcomes were evaluated with the Kaplan-Meier method and compared using the Log-Rank test. Multivariate Cox proportional hazards regression analysis was performed with statistically significant variables in the univariate analyses to assess the relationships between different variables and DFS or OS. The hazard ratios (HRs) and corresponding 95% confidence intervals (95%CI) were reported. All the statistical analyses were performed in R (version 4.0.4). *P* < 0.05 was considered statistically significant in a two-tailed test.

## Results

### Baseline characteristics

A total of 2070 patients met the selection criteria, including 207 patients in the S4RD− group and 1863 patients in the S4RD+ group. All patients were followed up in the clinic; however, 312 patients (15.1%) were lost during the follow-up period (36 patients, 17.4%, in the S4RD− group and 276 patients, 14.8%, in the S4RD+ group).

**Fig. 1:**
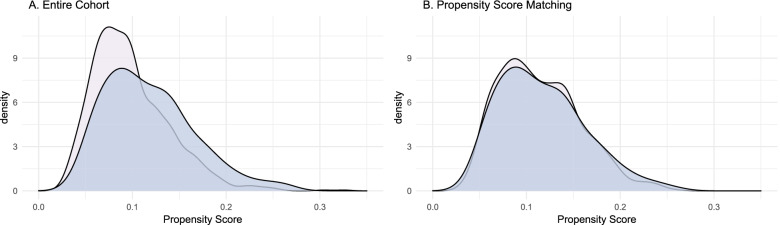
Density maps of propensity score of entire cohort (**A**) and propensity score matching (**B**)

There were statistically significant differences in the baseline smoking history (*P* < 0.001) and tumor location (*P* = 0.025) between the S4RD− and S4RD+ groups (Table 1). After the PSM, the score gap between the two groups decreased (Fig. 1). The smoking history (*P* = 0.926) and tumor location (*P* = 0.710) were also similar between the two groups (Table 1). Finally, the data for 1004 patients (205 in the S4RD− group and 799 in the S4RD+ group) were included in the outcome analysis.

**Table 1:** Baseline characteristics before and after propensity score matching

Variables	Entire cohort			Propensity score matching		
	S4RD− no. (%)	S4RD+ no. (%)	*P*	S4RD− no. (%)	S4RD+ no. (%)	*P*
No.	207	1863		205	799	
Age (year)			0.860			0.945
≥ 65	63 (30.4)	551 (29.6)		63 (30.7)	250 (31.3)	
< 65	144 (69.6)	1312 (70.4)		142 (69.3)	549 (68.7)	
Gender			0.368			0.808
Male	109 (52.7)	1047 (56.2)		107 (52.2)	407 (50.9)	
Female	98 (47.3)	816 (43.8)		98 (47.8)	392 (49.1)	
Smoking history			< 0.001			0.926
Yes	92 (44.4)	1080 (58.0)		91 (44.4)	360 (45.1)	
No	115 (45.6)	783 (42)		114 (55.6)	439 (54.9)	
Surgical approaches			0.791			0.585
Thoracotomy	114 (55.1)	1003 (53.8)		112 (54.6)	417 (52.2)	
Thoracoscopic surgery	93 (44.9)	860 (46.2)		93 (45.4)	382 (47.8)	
Tumor location			0.025			0.710
Upper lobe	91 (44.0)	645 (34.6)		97 (47.3)	373 (46.7)	
Middle lobe	19 (9.2)	224 (12.0)		19 (9.3)	90 (11.3)	
Lower lobe	97 (46.9)	994 (53.4)		89 (43.4)	336 (42.1)	
Histological type			0.065			0.601
Adenocarcinoma	157 (75.8)	1265 (67.9)		155 (75.6)	630 (78.8)	
Squamous cell carcinoma	33 (15.9)	397 (21.3)		33 (16.1)	113 (14.1)	
Others	17 (8.2)	201 (10.8)		17 (8.3)	56 (7.0)	
Adjuvant chemotherapy			0.218			0.415
No	140 (67.6)	1174 (63.0)		140 (68.3)	519 (65.0)	
Yes	67 (32.4)	689 (37.0)		65 (31.7)	280 (35.0)	
pT			0.436			0.943
T1a	23 (11.1)	169 (9.1)		23 (11.2)	88 (11.0)	
T1b	59 (28.5)	557 (29.9)		59 (28.8)	240 (30.0)	
T1c	56 (27.1)	524 (28.1)		56 (27.3)	224 (28.0)	
T2a	27 (13.0)	252 (13.5)		27 (13.2)	110 (13.8)	
T2b	17 (8.2)	157 (8.4)		17 (8.3)	49 (6.1)	
T3	13 (6.3)	147 (7.9)		13 (6.3)	56 (7.0)	
T4	12 (5.8)	57 (3.1)		10 (4.9)	32 (4.0)	
pN			0.058			0.565
N0	162 (78.3)	1355 (72.7)		162 (79.0)	628 (78.6)	
N1	15 (7.2)	109 (5.9)		14 (6.8)	42 (5.3)	
N2	30 (14.5)	399 (21.4)		29 (14.1)	129 (16.1)

### Involvements of mediastinal LN

**Fig. 2:**
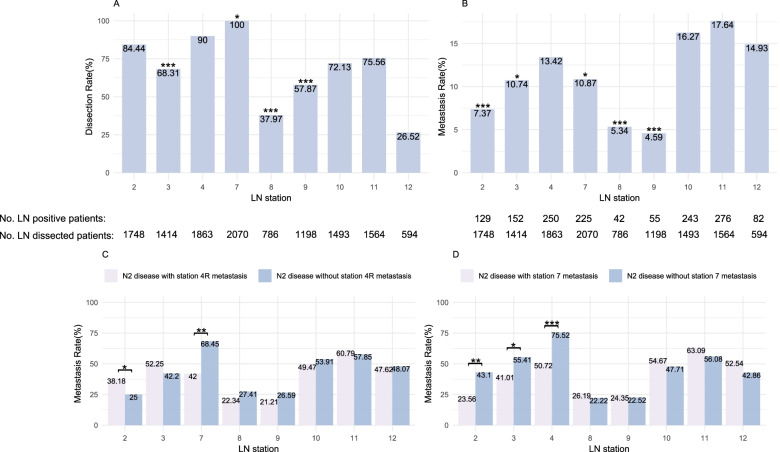
Dissection rate (**A**) and metastasis rate (**B**) of each lymph node station. Distribution of lymph node involvements in patients with N2 disease and station 4R (**C**) or 7 (**D**) metastasis. Chi-square analysis was used to compare the dissection or metastasis rates between subgroups. *P < 0.05, **P < 0.01, ***P < 0.001

The resection rate and metastasis rate of each LN station for all patients are shown in Fig. 2. All the station 7 LNs and 90.0% (1863/2070) of station 4R LNs were removed. The metastasis rate of station 4R was 13.4% (250/1863), which was significantly higher than the rates for all other stations, namely station 7 (10.9%, 225/2070, *P* = 0.034), station 3 (10.7%, 152/1414, *P* = 0.046), station 2 (7.4%, 129/1748, *P* < 0.001), station 8 (5.3%, 42/786, *P* < 0.001), and station 9 (4.6%, 55/1198, *P* < 0.001). In the S4RD+ group, subgroup analyses were performed based on the LN metastasis in stations 4 and 7 in patients with N2 diseases (Fig. 2C, D). The results showed that the metastatic rate of station 2 in patients with station 4R metastasis was significantly higher than that in patients without station 4R metastasis (38.2% vs. 25.0%, *P* = 0.017). The metastasis rate of station 7 in patients with station 4R metastasis was significantly lower than that in patients without station 4R metastasis (42.0% vs. 68.5%, *P* = 0.002). The metastasis rates of station 2, station 3, and station 4 in patients with station 7 metastasis were lower than those in patients without station 7 metastasis (23.6% vs. 43.1%, *P* = 0.008, 41.0% vs. 55.4%, *P* = 0.020, and 50.7% vs. 75.5%, *P* < 0.001, respectively). In addition, in N2 patients with station 10 metastasis, the metastasis rate of station 4R (60.0%) was statistically significantly higher than that of station 2 (34.3%, *P* < 0.001), station 3 (44.7%, *P* = 0.018), station 7 (52.9%, *P* = 0.252), station 8 (33.3%, *P* < 0.001), and station 9 (18.0%, *P* < 0.001).

### Variables associated with station 4R metastasis

Univariate and multivariate analysis demonstrated that tumor location (*P* = 0.013) and pT stage (*P* < 0.001) were significantly associated with station 4R metastasis, whereas gender, age, smoking history, surgical approaches, and pathological types had no relationship with station 4R metastasis (Table 2). In addition, station 2 (*P* < 0.001), station 3 (*P* < 0.001), station 7 (*P* < 0.001), and station 11 (*P* < 0.001) metastases were associated with station 4R metastasis, whereas station 8 (*P* = 0.432) and 9 (*P* = 0.355) metastases were not associated with station 4R metastasis.

**Table 2:** Univariate and multivariate analyses of variables associated with station 4R lymph node metastasis

Variables	No.	Univariate analyses			Multivariate analyses		
		Station 4R metastasis no. (%)		*P*			
		Positive	Negative		OR	95% CI	*P*
Age (year)				0.208			
< 65	1312	185 (14.1)	1127 (85.9)				
≥ 65	551	65 (11.8)	486 (88.2)				
Gender				0.273			
Female	816	101 (12.4)	715 (87.6)				
Male	1047	149 (14.2)	898 (85.8)				
Smoking history				1.000			
Yes	1080	145 (13.4)	935 (86.6)				
No	783	105 (13.4)	678 (86.6)				
Surgical approaches				< 0.001			
Thoracoscopic surgery	860	78 (9.1)	782 (90.9)		1		
Thoracotomy	1003	172 (17.2)	831 (82.8)		1.19	0.81–1.77	0.362
Tumor location				0.013			
Upper lobe	994	154 (15.5)	840 (84.5)		1		
Middle lobe	224	21 (9.4)	203 (90.6)		0.46	0.24–0.84	0.016
Lower lobe	645	75 (11.6)	570 (88.4)		0.44	0.29–0.65	< 0.001
Histological type				0.305			
Adenocarcinoma	1265	178 (14.1)	1087 (85.9)				
Squamous cell carcinoma	397	44 (11.1)	353 (88.9)				
Others	201	28 (13.9)	173 (86.1)				
pT stage				< 0.001			
T1a	169	8 (4.7)	161 (95.3)		1		
T1b	557	52 (9.3)	505 (90.7)		1.73	0.73–4.63	0.241
T1c	524	79 (15.1)	445 (84.9)		2.79	1.21–7.38	0.025
T2a	252	39 (15.5)	213 (84.5)		2.65	1.06–7.39	0.047
T2b	157	26 (16.6)	131 (83.4)		2.36	0.86–7.05	0.106
T3	147	34 (23.1)	113 (76.9)		4.89	1.87–14.07	0.002
T4	57	12 (21.1)	45 (78.9)		4.17	1.28–14.05	0.019
Station 2 metastasis	116	84 (72.4)	32 (27.6)	< 0.001	11.19	6.67–19.06	< 0.001
Station 3 metastasis	139	93 (66.9)	46 (33.1)	< 0.001	6.06	3.79–9.72	< 0.001
Station 7 metastasis	207	105 (50.7)	102 (49.3)	< 0.001	5.02	3.31–7.63	< 0.001
Station 8 metastasis	38	21 (55.3)	17 (44.7)	< 0.001	1.43	0.59–3.48	0.423
Station 9 metastasis	53	28 (52.8)	25 (47.2)	< 0.001	1.44	0.65–3.13	0.355
Station 10 metastasis	220	93 (42.3)	127 (57.7)	< 0.001	2.15	1.41–3.24	< 0.001
Station 11 metastasis	248	107 (43.2)	141 (56.8)	< 0.001	2.58	1.71–3.87	< 0.001

### Survival outcomes

**Fig. 3:**
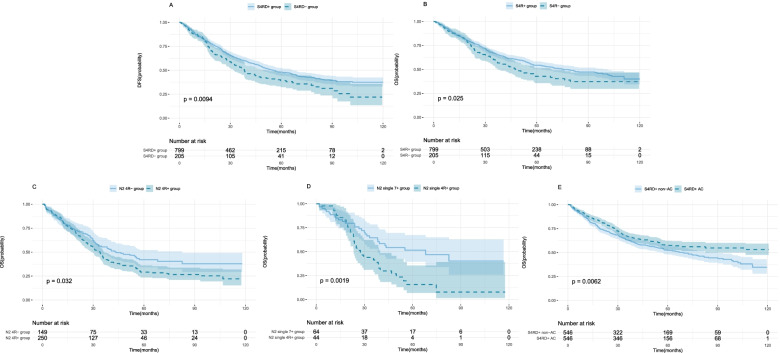
Kaplan–Meier curves for DFS (**A**) and OS (**B**) between propensity-matched N2 patients with or without station 4R dissection. **C** OS of patients without or with station 4R metastasis. **D** OS of patients with single-station 4R or station 7 metastasis. **E** OS of patients with adjuvant chemotherapy and station 4R dissection (S4RD+ AC) or not (S4RD− AC)

There were 1004 patients included in the survival analysis after PSM. In the end, 157 patients (15.6%) were lost during the follow-up period, including 36 patients (17.6%) in the S4RD− group and 121 patients (15.1%) in the S4RD+ group. The median follow-up periods were 64.9 and 59.7 months in the S4RD− and S4RD+ groups, respectively (*P* = 0.39). The 5-year DFS was 48.1% in the S4RD+ group and 49.1% in the S4RD− group. The 5-year OS was 54.4% in the S4RD+ group and 42.8% in the S4RD− group. The DFS and OS of patients with station 4R dissection were significantly higher than the values for those without 4R dissection (*P* = 0.009 and 0.025, respectively (Fig. 3A, B). Univariate analysis showed that station 4R dissection, pT stage, pN stage, histological type, and pTNM stage were related to the DFS and OS (Table 3). Multivariate analysis showed that station 4R dissection was an independent risk factor for DFS (HR 1.33, 95%CI 1.08–1.64, *P* = 0.007) and OS (HR 1.31, 95%CI 1.04–1.63, *P* = 0.018) (Table 3). After excluding patients with no station 4R dissection, further analysis of patients with N2 LN metastasis showed that the 5-year OS of the group with station 4R metastasis was significantly lower than that of the group without station 4R metastasis (250 vs.149 patients; 29.1% vs. 42.1%, *P* = 0.032; Fig. 3C). The 5-year OS of the single-station 4R metastasis group was significantly lower than that of the single-station 7 metastasis group (44 vs. 64 patients; 15.7% vs. 51.6%, *P* = 0.002, Fig. 3D). After re-performing PSM based on chemotherapy in patients with 4R station dissection, the analysis results showed that the prognosis of patients who received adjuvant chemotherapy was better than that of patients who did not (546 vs. 546 patients; 57.4 % vs. 52.3%, *P* = 0.006, Fig. 3E).

**Table 3:** Univariate and multivariate Cox regression analyses of prognostic factors in propensity-matched patients

Variables	DFS						OS					
	Univariate analyses			Multivariate analyses			Univariate analyses			Multivariate analyses		
	HR	95% CI	*P*	HR	95% CI	*P*	HR	95% CI	*P*	HR	95% CI	*P*
Age (year)												
< 65	1						1					
≥ 65	0.93	0.77–1.13	0.465				0.98	0.80–1.20	0.847			
Gender												
Female	1						1					
Male	1.09	0.91–1.30	0.309				1.15	0.96–1.39	0.131			
Smoking history												
No	1						1					
Yes	1.02	0.86–1.22	0.792				1.07	0.89–1.28	0.496			
Surgical approaches												
Thoracoscopic surgery	1						1					
Thoracotomy	1.084	0.91–1.29	0.366				1.15	0.95–1.39	0.146			
Tumor location												
Upper lobe	1						1					
Middle lobe	0.99	0.74–1.33	0.952				1.09	0.79–1.48	0.607			
Lower lobe	1.06	0.88–1.28	0.514				1.12	0.92–1.36	0.270			
Histological type												
Adenocarcinoma	1						1					
Squamous cell carcinoma	0.79	0.61–1.03	0.085				0.83	0.63–1.09	0.182			
Others	1.00	0.72–1.38	0.992				1.06	0.76–1.49	0.719			
Adjuvant chemotherapy												
No	1						1					
Yes	0.97	0,81–1.17	0.75				1.01	0.83–1.23	0.926			
pT stage												
T1a	1			1			1			1		
T1b	0.90	0.65–1.26	0.569	0.92	0.66–1.28	0.631	0.98	0.68–1.40	0.917	0.98	0.68–1.42	0.950
T1c	1.23	0.89–1.70	0.191	1.16	0.84–1.61	0.341	1.29	0.90–1.84	0.152	1.20	0.84–1.72	0.307
T2a	1.27	0.89–1.82	0.184	0.91	0.48–1.73	0.795	1.46	0.99–2.15	0.055	1.05	0.55–2.03	0.862
T2b	1.49	0.98–2.28	0.061	1.13	0.60–2.13	0.684	1.73	1.10–2.71	0.017	1.21	0.62–2.35	0.571
T3	1.23	0.81–1.88	0.322	1.43	0.47–4.31	0.521	1.44	0.91–2.26	0.110	1.81	0.56–5.75	0.315
T4	1.96	1.25–3.09	0.003	1.44	0.33–6.31	0.622	2.13	1.31–3.48	0.002	1.84	0.40–8.42	0.428
pN stage												
N0	1			1			1			1		
N1	1.70	1.19–2.42	0.003	2.16	0.86–5.39	0.097	1.83	1.27–2.63	0.001	2.55	0.98–6.66	0.054
N2	1.83	1.48–2.27	< 0.001	1.65	0.29–9.24	0.568	1.93	1.54–2.42	< 0.001	2.25	0.36–13.91	0.382
Station 4R LN dissection												
Yes	1			1			1			1		
No	1.31	1.06–1.61	0.009	1.33	1.08–1.64	0.007	1.28	1.03–1.60	0.025	1.31	1.04–1.63	0.018
pTNM stage												
IA	1			1			1			1		
IB	1.33	1.01–1.76	0.044	1.48	0.78–2.81	0.228	1.45	1.08–1.95	0.013	1.46	0.76–2.81	0.251
IIA	1.46	0.96–2.22	0.074	1.29	0.63–2.62	0.474	1.66	1.08–2.56	0.021	1.44	0.69–3.00	0.327
IIB	1.27	0.94–1.72	0.116	0.76	0.28–2.07	0.595	1.41	1.02–1.93	0.034	0.69	0.24–1.99	0.502
IIIA	1.87	1.49–2.35	<0.001	1.09	0.19–6.09	0.913	2.00	1.57–2.54	<0.001	0.85	0.13–5.21	0.863
IIIB	2.78	1.78–4.35	<0.001	1.24	0.06–23.72	0.884	3.07	1.96–4.82	<0.001	0.82	0.03–18.11	0.902

## Discussion

Mediastinal LN dissection is an essential step during lung cancer surgical treatment. It plays an important role in the therapeutic and postoperative staging of lung cancer. Evaluation of metastasis in the mediastinal LN can also provide information on the prognosis of patients, as well as guide further treatment [15, 16]. However, at the present time, there is no conclusive evidence to support the dissection of station 4R during right lung cancer treatment [17–19]. The relevant guidelines also do not provide any recommendations on the extent of dissection [6]. In a previous study in patients who received lobectomy with MLND due to primary non-small cell lung cancer with a diameter smaller than 3 cm, the station 4R resection rates were 69%, 63%, and 61% in patients with the upper, middle, and lower lobe lesions, respectively [10]. Liu et al. reported a station 4R resection rate of 87% in patients who underwent lobectomy with MLND [8]. A Canadian survey of 86 thoracic surgeons found that 5 to 30% of surgeons did not consider that the station 4R dissection was necessary for different tumor sites [9]. In another study on patients with the lobe-specific lymph node dissection, the resection rate of station 4R in patients with right upper lobe lung cancer was only 70%, even if the station 4R metastasis rate was high up to 21% [11]. All this evidence suggested that a significant number of patients with the station 4R metastases might not be managed properly. Their managements could be delayed due to the errors in the pathological staging. The station 4R metastasis rate was the highest, or second only to that of station 7, in patients with right primary NSCLC [8, 10]. Therefore, we performed this retrospective study to explore the prognostic significance of station 4R dissection in patients with right primary NSCLC.

The results of our study suggested that the most common mediastinal LN station with metastasis was station 4R. We further analyzed LN metastasis in N2 patients with station 4R dissection in the subgroup of patients with and without station 4R or station 7 metastasis. The results indicated that the metastatic rate of station 2 was significantly higher in the group with station 4R metastasis than in the group without station 4R metastasis, whereas the metastasis rate of station 7 was lower in the group with station 4R metastasis than in the group without station 4R metastasis. The metastatic rates of stations 2, 3, and 4 were significantly lower in the group with station 7 metastasis than in the group without station 7 metastasis. There were no statistically significant differences in the metastasis rates among stations 8, 9, 11, and 12 in the subgroup analyses. N2 patients with station 10 metastasis had the highest station 4R metastasis rate, suggesting a positive correlation between the metastases of station 10 and station 4R. The anatomical associations among stations 2, 3, 4, and 10 might contribute to the difference in the metastases rates among the above subgroups. Univariate analysis suggested that the tumor location, surgical approaches, pT stage, and other LN station metastases were associated with station 4R metastasis. Multivariate analysis showed that the tumor location, pT stage, and stations 2, 3, 7, 10, and 11 metastases were independent risk factors for station 4R metastasis. The anatomical interactions among these LNs might contribute to the close association of metastases among them. When there was metastasis in stations 2, 3, 7, 10, and 11, the cancer cells could migrate to station 4 through the lymphatic duct, resulting in metastasis to the other station [20]. Riquet et al. [21] proposed that stations 2, 4, and 10 had the same drainage pathway and that tracheoesophageal LNs in station 3 were closely associated with the right paratracheal LNs. Zheng et al. [22] suggested that the 3A LN participated in the mediastinal lymphatic drainage system due to an extensive communication network among stations 4R, 2R, and 10R. Our study also suggested that stations 7 and 11 were independently associated with station 4R metastasis, although there was no strong evidence to support an anatomical connection among these stations. We also found that the tumor location was an independent risk factor for station 4R metastasis. The risk of station 4R metastasis was higher with upper lobe tumors than with middle and lower lobe tumors, which was consistent with many previous studies. Upper lobe NSCLCs are more likely to metastasize to the mediastinal LN, which is the underlying theory for dedicated lung lymphadenectomy [10, 23, 24]. Our results also suggested that the pT stage was a risk factor for station 4R metastasis, which was in agreement with other studies showing a relationship between tumor size and mediastinal LN metastasis [25, 26]. Zhang et al. suggested that systematic LN dissection should be performed, since tumor size was an independent risk factor for LN metastasis in patients even with pT1 stage lung adenocarcinoma [27]. Similarly, Jia et al. proposed that the tumor volume, but not the maximum diameter, could be the indicator of the tumor burden. A larger tumor usually correlated with a high risk of LN metastasis [28]. Other studies have indicated that the preoperative carcinoembryonic antigen [29], platelet count [30], and plasma D-dimer level [31] could be used to predict LN metastatic status in patients with operable NSCLC, suggesting that surgeons should carefully review the imaging and laboratory results and make comprehensive preoperative evaluations on the possibility of LN metastasis in NSCLS patients.

The results of survival analysis showed that DFS and OS in the S4RD+ group were significantly higher than those in the S4RD− group. Multivariate analysis suggested the status of station 4R dissection was an independent risk factor for DFS and OS. The station 4R metastasis rate was the highest among all mediastinal LN stations. Patients without station 4R dissection could have lower postoperative pathological staging and therefore might receive inadequate postoperative treatments. MLND could increase the accuracy of staging by improving the detection of occult N2 disease [17, 32]. Thorough MLND could improve the survival chances of patients. In addition, station 4R dissection could facilitate the clearance of potential LN micrometastases and thus block the development of LN metastases at an early stage [33]. These were consistent with previous studies on the single LN station [8, 34]. In other studies on the number of LN dissection, the researchers also believed that a high number of LN dissection was associated with improved OS and DFS [35, 36]. More LN dissections could make a higher accurate TNM staging and would be more likely to remove occult metastasis. A small number of dissected LN could make the N staging difficult [37]. A high accurate TNM staging is essential to determine the appropriate adjuvant therapy strategies and improve the survival chance. The number of LN removed and examined is determined by the different surgical operative approaches. A meta-analysis reported that, compared with the thoracoscope surgery, the thoracotomy could remove more numbers of total LN and N2 LN, although both surgery approaches removed similar numbers of N1 LN [38]. Our current study did not show a difference between different approaches. Future higher quality and large-scale randomized controlled trials are required to confirm this.

We further performed subgroup survival analyses after excluding patients without station 4R dissection. Patients were assigned into two subgroups based on the status of station 4R metastasis, single-station 4R metastasis, or single-station 7 metastasis. The results showed that the prognosis was worse in the group with station 4R metastases than in the group without station 4R metastases. The prognosis of the single-station 4R metastasis group was worse than that of the single-station 7 metastasis group. Of note, our univariate analysis did not support an association between adjuvant chemotherapy and the outcomes in 1004 PSM patients. This might be due to the imbalanced baseline characteristics, pT stage, and pN stage between patients with and without chemotherapy. When we analyzed this association in all the enrolled PSM patients, patients who received chemotherapy had a higher OS compared to patients who did not. Adjuvant chemotherapy was a risk factor for the treatment prognosis in patients with station 4R dissection.

In summary, we demonstrated the importance of station 4R LNs based on studies on LN metastasis and patient prognosis. Station 4R dissection should be a required step during the MLND in patients with right NSCLC. In clinical practice, patients were more likely to receive station 7, but not station 4R, dissection. Our results suggested that station 4R was more important than station 7 in terms of patient outcomes. Recent advances in endoscopic and robotic techniques could facilitate the dissection and removal of station 4R with complex anatomical structures. Finally, we recommend that station 4R dissection should be a routine procedure during MLND for patients with right lung cancer.

Our present study showed that the LN dissection was correlated with the outcomes in patients with NSCLC. LN metastasis and dissection in different locations could also have different impacts on the survivals. A recent study proposed a nomogram that could predict the survival in patients with stage III N2 small cell lung cancer. The nomogram also included the LN metastasis [39]. Whether different locations of the LN metastasis could have different influences on the survivals in patients with small cell lung cancer requires further studies.

The limitations of the current study included its retrospective and single-center design, which could introduce selection bias in the analysis. We performed PSM during the analyses for all enrolled patients, but not in the subgroup due to a relatively small number of patients. This might fail to reduce the data bias and confounding variables, resulting in systemic bias. In addition, a total of 312 (15.1%) patients were lost during the follow-up period, which might cause bias in survival analysis. Therefore, further multicenter randomized clinical trials are required to confirm our study results.

## Conclusion

Station 4R had the highest metastasis rate among all the mediastinal stations in patients with right primary NSCLC. These patients could benefit from station 4R dissection to have a better survival chance. Routine station 4R dissection should be recommended during surgical treatment for these patients.

## Data Availability

The datasets generated and analyzed during the current study are not publicly available due to none of the data types requiring uploading to a public repository but are available from the corresponding author on reasonable request.

## Abbreviations

NSCLC: Non-small cell lung cancer
DFS: Disease-free survival
OS: Overall survival
S4RD− group: Patients having no station 4R dissection
S4RD+ group: Patients having station 4R dissection
MLND: Mediastinal lymph node dissection
LNs: Lymph nodes
IASLC: International Association for the Study of Lung Cancer
CT: Computed tomography
MRI: Magnetic resonance imaging
PET/CT: Positron emission tomography–computed tomography
PSM: Propensity score matching
HRs: Hazard ratios
95%CI: 95% confidence intervals
